# 

**Published:** 1907-10

**Authors:** 


### The American Journal of Dental Science

The Official Organ of the Florida, Georgia and South Carolina State Dental Societies

**Third Series**

VOL. XXXVIII OCTOBER, 1907 NO. 10

EDITORS

WM. GIRD BEECROFT, D. D. S., Madison, Wis. B. H. TEAGUE, D. D. S., Aiken, S. C

Published on the 10th day of each month.
Subscription price $1.00 per year.
ENTERED AS SECOND-CLASS MATTER, MADISON, WIS.

Address all communications, both business and editorial, to
**The Dental Publishing Company, Madison, Wisconsin**

### MEETINGS.

The next meeting of the Nebraska State Dental Board will be held at the State House, Lincoln, Nebr., Nov. 18th, 19th and 20th, 1907. All applications for examination must be in the hands of the secretary at least five days before this date. For any further information address,

C. F. LADD, Sec. Nebraska State Dental Board.

